# miRNAs in Urine Extracellular Vesicles as Predictors of Early-Stage Diabetic Nephropathy

**DOI:** 10.1155/2016/7932765

**Published:** 2016-01-28

**Authors:** Yijie Jia, Meiping Guan, Zongji Zheng, Qian Zhang, Chuan Tang, Wenwei Xu, Zhizhou Xiao, Ling Wang, Yaoming Xue

**Affiliations:** Department of Endocrinology & Metabolism, Nanfang Hospital, Southern Medical University, Guangzhou, Guangdong 510515, China

## Abstract

*Background*. miR-192, miR-194, and miR-215 are enriched in the kidney and play roles in the pathogenesis of diabetic nephropathy (DN). Extracellular vesicles (EVs) can be detected in body fluids and may serve as disease biomarkers.* Methods*. Eighty type 2 diabetes patients with normoalbuminuria (*n* = 30), microalbuminuria (*n* = 30), or macroalbuminuria (*n* = 20), as well as 10 healthy controls, were enrolled in this study. Real-time PCR was used to evaluate urinary EV miRNAs expression.* Results*. The miR-192 levels were significantly higher than the miR-194 and miR-215 levels in urine EVs and all three miRNAs were significantly increased in the microalbuminuric group compared with the normoalbuminuric and control subjects but were decreased in the macroalbuminuric group. In patients with normoalbuminuria and microalbuminuria, miR-192 was positively correlated with albuminuria (*r* = 0.357, *P* = 0.005) levels and transforming growth factor- (TGF-) *β*1 (*r* = 0.356, *P* = 0.005) expression. Receiver operating characteristic (ROC) curve analysis revealed that miR-192 was better than miR-194 and miR-215 in discriminating the normoalbuminuric group from the microalbuminuric group. Exposure of human renal tubular epithelial cells to high glucose increased the expression of both miRNAs in cellular supernatant EVs, indicating a potential source.* Conclusion*. These results suggest the potential use of urinary EV miR-192 as a biomarker of the early stage of DN.

## 1. Introduction

Diabetic nephropathy (DN) occurs in over 30% of patients with type 2 diabetes mellitus (T2DM) and is the main cause of end-stage renal disease (ESRD) [1]. The stages of DN are defined as incipient, manifest, and advanced, and DN usually develops over a period of years [2, 3]. Identifying patients in the early stage of DN is an important step for effective management and treatment [4].

MicroRNAs (miRNAs) are a group of short (~21 nucleotides) noncoding RNAs that are critically involved in many biological processes and may serve as potential biomarkers of several diseases due to their specificity and stability in body fluids [5, 6]. miR-192, miR-194, and miR-215 have been reported to be highly expressed in kidney tissue and play critical roles in kidney development and differentiation [7]. miR-215 and miR-194-1 are located on the same chromosome, as are miR-192 and miR-194-2. miR-192 and miR-215 share the same seed sequence, and miR-194-1 and miR-194-2 possess the same mature sequence [8]. miR-192 and miR-215 have been shown to be dramatically upregulated and are involved in promoting renal glomerular and tubulointerstitial fibrosis in DN [9]. However, in another study, miR-192 and miR-194 were found to be decreased in the mesangial cells of diabetic mice [10]. Little is known regarding the changes of these three miRNAs in urine during the development of DN and their correlation with kidney function.

Extracellular vesicles (EVs) are divided into two groups according to their diameter: microvesicles (100–1000 nm) and exosomes (30–150 nm) [11, 12]. EVs are released by different cell types and can be isolated from body fluids, including plasma, urine, milk, and saliva [13, 14]. Recently, EVs have been found to contain proteins, mRNAs, and miRNAs that may influence cell-to-cell communication [15, 16]. Urinary EVs are released from all nephron segments, reflecting the progress of renal damage, and could be used as a “fluid biopsy” [17]. Both Lv et al. [18] and Solé et al. [19] reported that urinary exosomal miR-29c levels are correlated with the degree of histological fibrosis, indicating that exosomal miRNAs could be used as novel noninvasive markers of kidney disease.

In this study, we compared the levels of miR-192, miR-194, and miR-215 isolated from urinary EVs from T2DM patients with different degrees of albuminuria and examined whether the levels of these miRNAs correlated with clinical parameters to determine whether urinary EV miRNAs can serve as early biomarkers of DN.

## 2. Material and Methods

A total of 80 diagnosed T2DM patients admitted to the Department of Endocrinology & Metabolism, Nanfang Hospital, Southern Medical University, between October 2014 and May 2015, were enrolled in this study. According to the degree of albuminuria, patients were classified into a normoalbuminuric group (ACR < 2.5 mg/mmol, *N* = 30), a microalbuminuric group (ACR between 2.5 mg/mmol and 25 mg/mmol, *N* = 30), and a macroalbuminuric group (ACR > 25 mg/mmol, *N* = 20). Ten age matched healthy volunteers were randomly selected from the Center for Health Promotion at Nanfang Hospital. The exclusion criteria were the following: active urinary tract infection, nondiabetic kidney diseases, neoplastic disorders, severe liver disease, inflammatory disorders, pregnancy, and a recent history of acute myocardial infarction, stroke, or occlusive peripheral vascular disease. Patients with an eGFR < 15 mL/min/1.73 m^2^ and those with macroalbuminuria/microalbuminuria who did not present with diabetic retinopathy were excluded. This study was approved by the ethics committee of Nanfang Hospital, Southern Medical University. All patients provided signed informed consent prior to participating in the study.

Patient demographics and clinical characteristics were obtained from medical records, including age, sex, duration of diabetes, systolic blood pressure (SBP), diastolic blood pressure (DBP), glycosylated hemoglobin (HbA1C), serum creatinine, cystatin, cholesterol (TC), triglycerides (TGs), low-density lipoprotein-cholesterol (LDL-C), high-density lipoprotein-cholesterol (HDL-C), urinary albumin excretion rate (UAER), and urinary albumin/creatinine ratio (UACR). Body mass index (BMI) was calculated as weight (kg)/height (m)^2^, and eGFR was estimated according to the Chronic Kidney Disease Epidemiology Collaboration (CKD-EPI) creatinine equation (http://www.qxmd.com/calculate-online/nephrology/ckd-epi-egfr).

### 2.1. Urine EVs Isolation

Initially, 50 mL of first-morning urine was collected from all subjects in sterile containers. All urine samples were processed within 1 h. Urinary cells were removed by centrifugation at 300 ×g (Rotor: JA-20; Beckman Coulter, Fullerton, CA, USA) for 15 min at 4°C, followed by centrifugation at 17,000 ×g (Rotor: JA-20; Beckman Coulter, Fullerton, CA, USA) for 15 min at 4°C and ultracentrifugation at 170,000 ×g for 70 min at 4°C (Rotor: SW 32Ti; Beckman Coulter, Brea, CA, USA). Pellets were washed using 8 mL of sterile phosphate-buffered saline (PBS) and ultracentrifuged at 170,000 ×g for 70 min. Subsequently, pellets were suspended in 100 *μ*L of PBS and were stored at −80°C for further analyses.

### 2.2. Cell Culture and EVs Extraction

The human renal tubular epithelial cell line HK-2 (ATCC, Manassas, USA) and the human mesangial cell line hMC (ScienCell, San Diego, USA)were cultured in Dulbecco's modified Eagle's medium, containing 5.5 mM D-glucose and 10% fetal bovine serum (FBS; Gibco, Australia), in a 5% CO_2_ incubator at 37°C until they reached 60% confluency. For EVs isolation, cells were cultured in medium containing 5.5 mM or 30 mM D-glucose supplemented with 2% exosome-depleted FBS for 48 h. Bovine exosomes were removed by ultracentrifugation at 170,000 ×g for 16 h at 4°C [20]. Cell supernatants were collected, and EVs were isolated by differential centrifugations as described previously.

### 2.3. Transmission Electron Microscopy

Samples were processed fresh for use in electron microscopy. Pellets were loaded onto 200-mesh nickel grids for three minutes at RT, and excess fluid was removed. Each grid was transferred to a drop of 2% phosphotungstic acid and incubated for 1 min at RT for staining. Phosphotungstic acid was removed, and the pellets were allowed to air-dry. Electron micrographs were collected using a Tecnai G2 Spirit electron microscope (FEI Company) for urinary pellets and a JEOL JEM-1400 for cellular pellets.

### 2.4. Western Blotting

Total protein levels in the EVs were measured using the BCA Protein Assay Kit (Takara Biotechnology), and an equal amount of protein (20 *μ*g) was loaded and separated by SDS-PAGE. Subsequently, the separated proteins were transferred onto polyvinylidene fluoride (PVDF) membranes (Merck Millipore, MA, USA). Membranes were blocked in Tris-buffered saline with 0.1% Tween-20 (TBST) containing 5% nonfat dry milk for 1 h. Membranes were incubated at 4°C overnight with an anti-CD63 primary antibody (sc-15363; Santa Cruz Biotechnology, Santa Cruz, CA, USA). Blots were washed in TBST and were then incubated with a fluorescent secondary antibody at room temperature for 1 h (1 : 15,000; LI-COR Biosciences, Lincoln, NE, USA). Fluorescence images were obtained using the Odyssey infrared imaging system (LI-COR, Lincoln, NE, USA).

### 2.5. RNA Isolation and Reverse-Transcription Quantitative RT-PCR

Total RNA was extracted using Trizol reagent (DingGuo, Beijing, China) in accordance with the manufacturer's instructions. EV samples were spiked with 25 fmol of cel-miR-39 (TIANGEN, Beijing, China) after a 5 min incubation in Trizol as previously described. To confirm that the RNA was confined to the EVs, urinary EVs were treated with 0.1 *μ*g/*μ*L RNase A (Biosharp) for 10 min at 37°C. The RNA quantification was performed using a NanoDrop ND-1000 spectrophotometer (Thermo Fisher Scientific, Wilmington, DE, USA).

cDNA was synthesized using the Takara Prime Script® RT reagent kit (Takara, Dalian, China) according to the recommended protocols. qRT-PCR was performed using SYBR Green, and the results were normalized to glyceraldehyde 3-phosphate dehydrogenase (GAPDH) expression. Target mRNA relative values were expressed as 2^−ΔCT^. Primers for transforming growth factor- (TGF-) *β*1 and GAPDH were synthesized by Invitrogen (Shanghai, China) as follows: TGF-*β*11: forward 5′-GCCCTGGACACCAACTATTGC, reverse 5′-AGGCTCCAAATGTAGGGGCAGG; GAPDH: forward 5′-GGAGCGAGATCCCTCCAAAAT, reverse 5′-GGCTGTTGTCATACTTCTCATGG.

Small RNAs were reverse transcribed using the miRcute miRNA first-strand cDNA synthesis kit (TIANGEN). The RT-PCR reactions were performed using the miRcute miRNA qPCR detection kit (TIANGEN). To quantify EV miRNA levels, a median normalization procedure was performed as previously reported [21]. Briefly, normalized CT value = raw average CT value − (spike in average CT value of the same sample − median spike in CT value). Target miRNA relative values were expressed as 2^−ΔCT^ after being normalized to cel-miR-39. Primers for miR-192, miR-194, and miR-215 were purchased from TIANGEN.

Both mRNA and miRNA reactions were run in triplicate, with no-template controls, and were performed using the LightCycler480 Real-Time PCR System (Roche; Hoffmann-La Roche Ltd., Basel, Switzerland).

### 2.6. Statistical Analysis

Statistical analysis was performed with SPSS version 19.0. All results are presented as means ± SE. Data were compared using Student's *t*-test, Mann-Whitney *U* test, and Spearman's rank order correlation, as appropriate. The diagnostic efficiencies of urinary EV miR-192, miR-194, and miR-215 for the detection of early-stage DN were compared by receiver operating characteristics (ROC) curve analysis. A CT value over 40 was defined as undetectable. *P* values below 0.05 were considered significant.

## 3. Results

The demographic and clinical parameters of the recruited subjects are summarized in Table 1. There were no significant differences in age, gender, BMI, HbA1C %, TC, HDL-C, LDL-C, eGFR, Scr, and duration of diabetes among the normo-, micro-, and macroalbuminuric groups. The cystatin (*t* = 2.680, *P* = 0.010) levels, the urine albumin-to-creatinine ratio (UACR; *t* = 4.681, *P* < 0.001), and the urinary albumin excretion rate (UAER; *t* = 9.417, *P* < 0.001) were higher in patients with microalbuminuria compared with those with normoalbuminuria. Patients with macroalbuminuria exhibited a higher SBP (*t* = 2.105, *P* = 0.041), a higher DBP (*t* = 2.312, *P* = 0.025), and a higher cystatin (*t* = 3.686, *P* = 0.001) relative to patients with normoalbuminuria. TGs (*t* = 3.671, *P* = 0.020 and *t* = 2.566, *P* = 0.016, resp.), UACR (*t* = 4.224, *P* < 0.001 and *t* = 3.890, *P* < 0.001, resp.), and UAER (*t* = 4.670, *P* < 0.001 and *t* = 4.301, *P* < 0.001, resp.) levels were higher in the macroalbuminuric diabetic group compared to the normoalbuminuric and microalbuminuric diabetic groups.

### 3.1. Confirmation and Characterization of EVs

**Figure 1:**
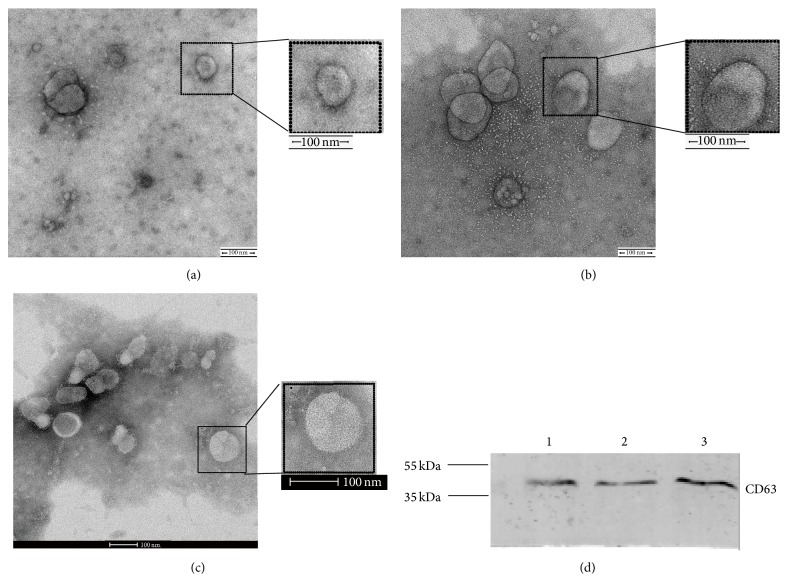
Characterization of EVs. (a) Transmission electron microscopy (TEM) of EVs from human proximal tubule cell supernatant. (b) TEM of EVs from mesangial cell supernatant. (c) TEM of urinary EVs. The scale bar represents 100 nm. (d) Western blot of EVs using the EV protein marker CD63. Lane 1: EVs from human proximal tubule cell supernatant; lane 2: EVs from mesangial cell supernatant; and lane 3: EVs from urine.

EVs isolated from urine samples and cell supernatants were examined using electron microscopy and found to be round structures with a size between 30 and 120 nm, consistent with previous reports [22]. Western blot analysis revealed the expression of CD63, a well-established marker [23] (Figure 1).

### 3.2. Expression of Urinary EV miRNAs

First, we measured miRNA levels in urinary EVs. As shown in Table 2, miR-192 levels were higher than the levels of miR-194 (*Z* = 7.913, *P* < 0.001) and miR-215 (*Z* = 8.405, *P* < 0.001) in urinary EVs. There was no significant difference between miR-194 and miR-215 (*Z* = 0.452, *P* = 0.651). The levels of these three miRNAs exhibited stepwise increases among healthy controls, the normoalbuminuric group, and the microalbuminuric group but decreases in the macroalbuminuric group. We further compared the expression of these three miRNAs in patients with normoalbuminuria (*n* = 30) and microalbuminuria (*n* = 30). Levels of miR-192 (*Z* = 3.777, *P* < 0.001), miR-194 (*Z* = 2.210, *P* = 0.027), and miR-215 (*Z* = 3.046, *P* = 0.002) in patients with diabetes with microalbuminuria were higher than those with normoalbuminuria.

### 3.3. Correlations between miRNAs and Renal Function in Patients with Normoalbuminuria and Microalbuminuria

**Figure 2:**
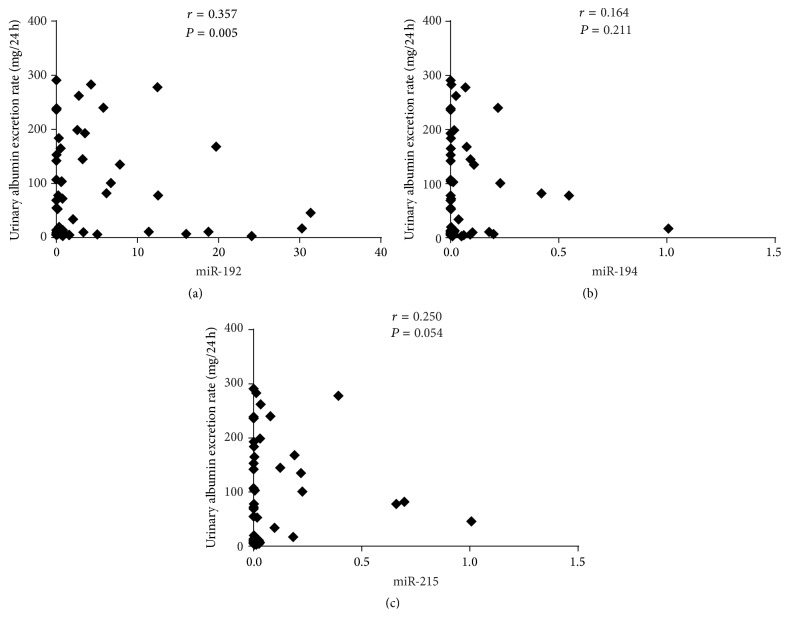
Correlation between urinary EV miRNAs levels and the urinary albumin excretion rate in T2DM patients with normoalbuminuria and microalbuminuria. A significant correlation between miRNA-192 levels and the UAER was detected. miRNA levels were normalized to cel-miR-39 and were calculated using the 2^−ΔCT^ method. miR-192 expression was significantly correlated with the urinary albumin excretion rate. Data were compared by Spearman's rank order correlations (*P* < 0.05).

**Figure 3:**
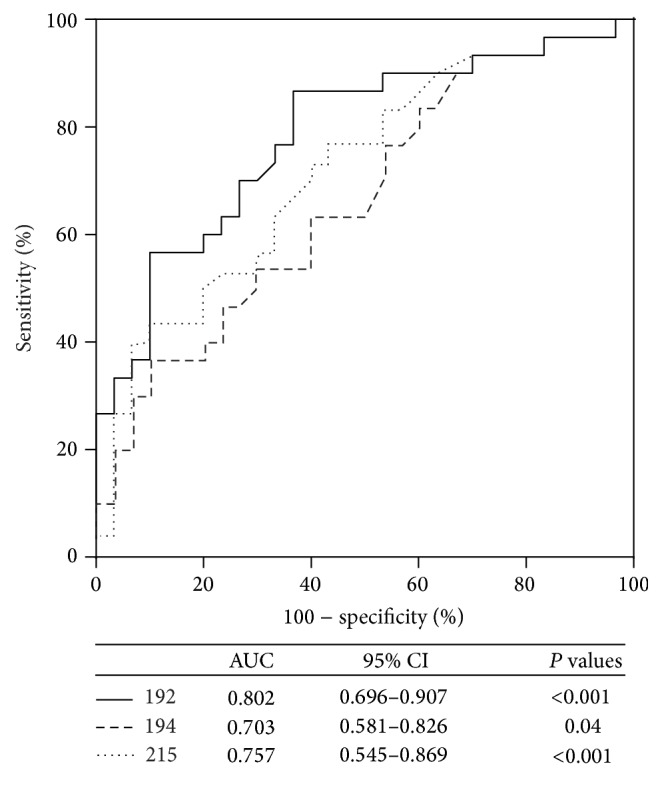
ROC analysis for miRNAs in patients with normoalbuminuria and microalbuminuria (AUC = 0.802, *P* < 0.001 for miR-192; AUC = 0.703, *P* = 0.04 for miR-194; and AUC = 0.757, *P* < 0.001 for miR-215).

We further analyzed the correlations between renal function parameters and the expression of miRNAs in patients with normoalbuminuria and microalbuminuria. As shown in Figure 2, UAER positively correlated with miR-192 expression (*r* = 0.357, *P* = 0.005). No correlation was found with miR-194 (*r* = 0.164, *P* = 0.211) and miR-215 (*r* = 0.250, *P* = 0.054). ROC analysis revealed that miR-192 had an area under the curve (AUC) of 0.802 (95% confidence interval, 0.696–0.907, *P* < 0.001), which was better than miR-194 with an AUC of 0.703 (95% confidence interval, 0.581–0.826, *P* = 0.04) and miR-215 with an AUC of 0.757 (95% confidence interval, 0.545–0.869, *P* < 0.001) in discriminating the normoalbuminuric group from the microalbuminuric group (Figure 3). For all three miRNAs, no significant correlation was detected with creatinine, cystatin, or eGFR (Table 3).

### 3.4. Correlations between miRNAs and TGF-*β*1 Expression in Patients with Normoalbuminuria and Microalbuminuria

**Figure 4:**
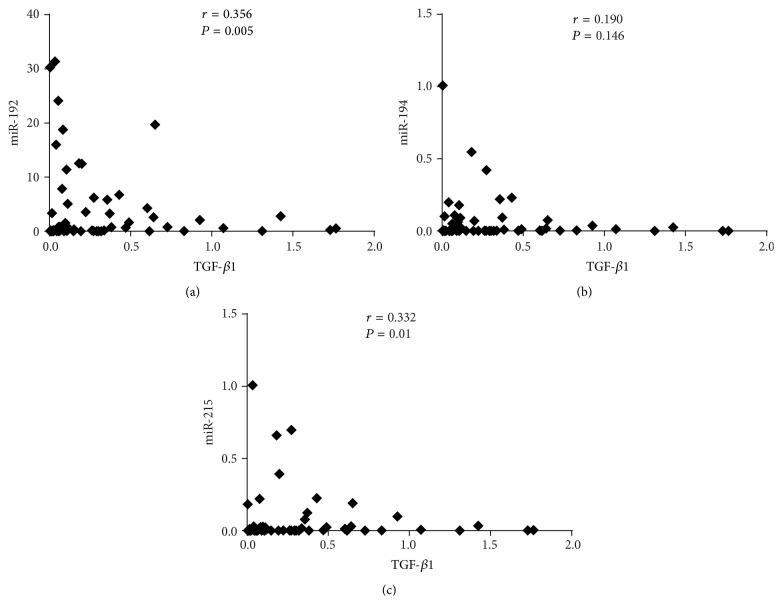
Urinary EV TGF-*β*1 expression and correlations with miRNAs. TGF-*β*1 levels were normalized to GAPDH and were calculated using the 2^−ΔCT^ method. (a–c) Correlation plots between miRNAs levels and TGF-*β*1 levels. miR-192 significantly correlated with TGF-*β*1 in urinary EVs. Data were compared by Spearman's rank order correlations (*P* < 0.05).

To investigate the correlations between the expression levels of these three miRNAs and TGF-*β*1, we measured the expression of TGF-*β*1 from urinary EVs in patients with normoalbuminuria and microalbuminuria. TGF-*β*1 levels were significantly correlated with miR-192 (*r* = 0.356, *P* = 0.005) and miR-215 (*r* = 0.332, *P* = 0.010), and no significant correlation was detected between TGF-*β*1 levels and miR-194 (*r* = 0.190, *P* = 0.146) (Figure 4).

### 3.5. miRNAs Expression in EVs from hMCs and HK-2 Cells Exposed to High Glucose

**Figure 5:**
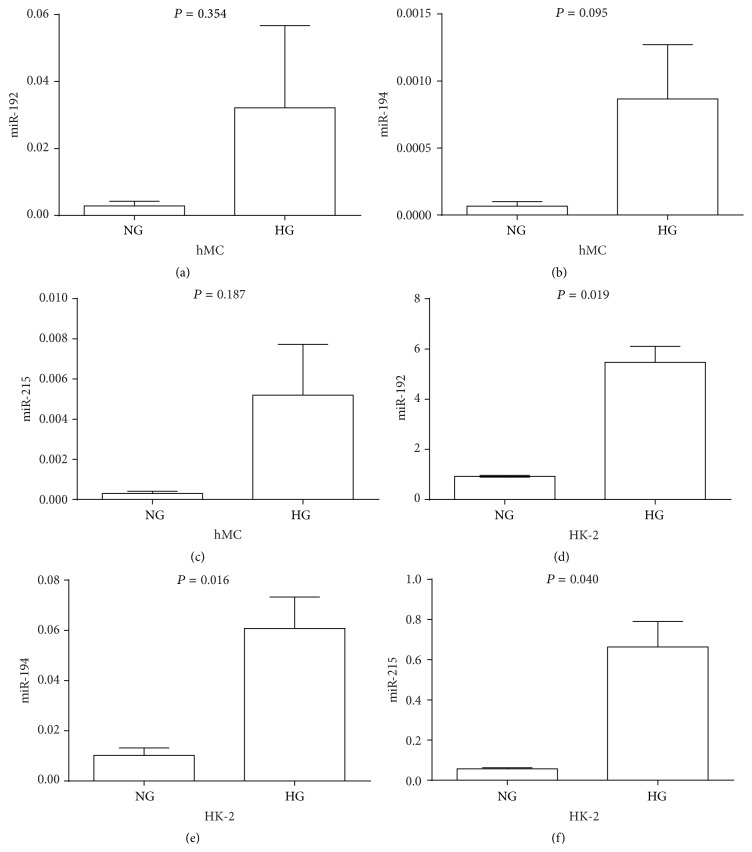
miRNAs expression from human proximal tubule and mesangial cell supernatant EVs. Human proximal tubule and mesangial cells were exposed to high glucose for 48 h. RT-PCR analysis was used to measure miRNA expression (a–c) from hMCs (d–f) from HK-2 cells. miRNAs levels were normalized to cel-miR-39 and were calculated using the 2^−ΔCT^ method (*P* < 0.05).

**Figure 6:**
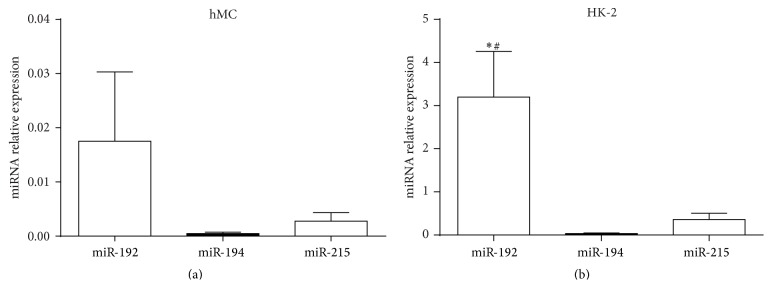
Comparison of the relative expression of miR-192, miR-194, and miR-215 in EVs from cellular supernatants from both the NG and HG groups. ^*∗*^The level of EV miRNA-192 compared with miR-194 (*P* < 0.05). ^#^The level of EV miR-192 compared with miR-215 (*P* < 0.05) (a) from hMCs and (b) from HK-2 cells. MiRNA levels were normalized to cel-miR-39 and calculated using the 2^−ΔCT^ method. Data are presented as the mean ± SE.

To measure whether exposure to high glucose could affect EV miRNAs expression in hMCs and HK-2 cells, we isolated EVs from the supernatants of both cell types. We found that exposure to high glucose significantly enhanced EV miR-192 (*t* = 7.129, *P* = 0.019), miR-194 (*t* = 4.008, *P* = 0.016), and miR-215 (*t* = 4.806, *P* = 0.04) expression levels in HK-2 cells (Figure 5). In addition, in EVs from HK-2 cells from both the NG and the HG groups, the miR-192 levels were significantly higher than those of miR-194 (*t* = 3.001, *P* = 0.03) and miR-215 (*t* = 2.668, *P* = 0.043). In EVs from hMCs, no significant differences were found between miR-192 and either miR-194 (*t* = 1.333, *P* = 0.212) or miR-215 (*t* = 1.146, *P* = 0.278) (Figure 6).

## 4. Discussion

miRNAs are important mediators in the development of DN and can be used as biomarkers to assess disease progression [24, 25]. Recent studies have demonstrated that EVs are enriched with miRNAs and that EV miRNAs are more stable than their cellular counterparts [26]. Previous studies have reported that urinary miRNAs are primarily detected in exosomes, and changes in miRNAs are significant in exosomes relative to other urine fractions [26], indicating that exosomal RNA in urine may serve as a potential renal disease marker.

miR-192, miR-194, and miR-215 are particularly abundant in the kidneys relative to other organs [7]. Furthermore, Saal and Harvey confirmed this expression pattern in rat kidneys and found that miR-192 and miR-194 are enriched in rat kidney cortex tissue relative to medulla tissue [27]. Recently, miR-192 and miR-194 were confirmed to be highly expressed in human proximal tubule and mesangial cells and to play key roles in acute kidney injury (AKI) [28]. In our study, these miRNAs were also detected in urinary EVs, and the relative abundance of miR-192 was significantly higher than those of miR-194 (*Z* = 7.913, *P* < 0.001) and miR-215 (*Z* = 8.405, *P* < 0.001), which suggests that miR-192 plays a more important role in the DN process, specifically in renal cell communication.

Previous studies of miRNA biomarkers of renal disease revealed that miRNA levels change throughout disease progression. Peng et al. [29] reported that miR-29a increased in T2DM patients with albuminuria. In addition to urinary sediment, Barutta et al. [30] reported that urinary exosomal miR-145 levels were increased whereas miR-155 and miR-424 were decreased in patients with microalbuminuria relative to those with normoalbuminuria. In our study, EV miRNAs expression increased in T2DM patients with microalbuminuria but decreased in patients with macroalbuminuria. In previous study miR-192 was found decreased in diabetic glomerulosclerosis patients with albuminuria between 3.5 and 9.7 g/d and eGFR between 87.8 and 5.6 mL/min/1.73 m^2^. This contradiction was also found in the study of miR-29a. In Peng et al.'s study [29] miR-29a was found increased in T2DM patients with albuminuria under 601.73 *μ*g/min and eGFR between 100.84 and 66.58 mL/min/1.73 m^2^. However in Lv et al.'s study [18] miR-29a was found decreased in CKD patients with albuminuria between 0.9 and 5.1 g/d and eGFR between 106.9 and 49.9 mL/min/1.73 m^2^. Neal et al. [31] reported that plasma-specific miRNAs decreased in patients with severe chronic renal failure compared with patients with mild renal impairment and mentioned that the decreasing of miRNAs may contribute to the pathogenesis of proteinuria and uremia. Therefore, we proposed that levels of albuminuria and eGFR can explain this contradiction. On the other hand, the expression of miR-192 in DN is controversial [32]; most investigations have found that miR-192 expression increases in DN models and in renal cells, but several other studies have reported that miR-192 expression decreases [33]. A recent study reported that miR-192 expression is higher in patients at the early stages of DN compared with those at the late stages [34]; however, in this study, they defined late stage as eGFR < 15 mL/min. As we did not enroll more patients with macroalbuminuria and cannot get biopsy from all patients, we cannot figure out miR-192 decreased at which point. These studies indicated that the increased expression of miR-192 was correlated to the early stage of DN and the controversy of miR-192 expression may contribute to the different stages of DN. Thus, we further analyzed the expression of miRNAs in T2DM patients with normoalbuminuria and microalbuminuria to identify the role of miRNAs as biomarkers at the early stages of DN.

We found that urinary EV miR-192 was positively correlated with albuminuria and can distinguish patients with normoalbuminuria from those with microalbuminuria (AUC = 0.802, *P* < 0.001). Thus, the large increase in EV miRNAs expression may have a potential use in the diagnosis of incipient DN. These results are consistent with the available literature, which suggests that miR-192 increases in the early stages of DN [33]. However, these results differ from the results of previous studies in which no significant differences in exosomal miR-192 expression were found between micro- and normoalbuminuric DM1 patients [30]. However, in the aforementioned previous study, U6 was used as an endogenous control. U6 has been reported to be unstable in circulation, and Hanke et al. [35] reported that U6 was not detectable in 8.5% of urine samples. This difference may also contribute to the different diabetes type. In that study, patients with type 1 diabetes mellitus (T1DM) were enrolled.

TGF-*β*1 is an important regulator of fibrosis during the process of DN and acts by inducing the expression of extracellular matrix (ECM) proteins [36]. Serum TGF-*β*1 has been shown to be increased in T2DM patients with microalbuminuria or macroalbuminuria relative to those with normoalbuminuria [37]. TGF-*β*1 has also been detected in urinary exosomes and was found to be increased in the urinary exosomes of patients with obstructive nephropathy [38]. Recent studies in DN mouse models have demonstrated that increased glomerular miR-192 levels are positively associated with renal TGF-*β*1 expression [39], and a feedback amplification circuit was detected between these two factors [40]. As we expected, TGF-*β*1 levels were positively correlated with both miR-192 (*r* = 0.356, *P* = 0.005) and miR-215 (*r* = 0.332, *P* = 0.010). These results suggest that urinary EVs may provide information that could indicate the pathological process underlying the early stages of DN.

Under various pathophysiological conditions, cells collectively package miRNA into EVs and release them into circulation [41]. Indeed, miR-192 and miR-194 are highly enriched in proximal tubule and mesangial cells [28], and miR-192 and miR-215 have been shown to play roles in mesangial cell hypertrophy and fibrogenesis [9, 42]. We hypothesized that these three miRNAs may serve as biomarkers of proximal tubule and mesangial cells in high-glucose conditions. In our study, we found that exposure to high glucose increased the expression of EV miR-192 and miR-215 in HK-2. In addition, we also determined that miR-192 expression was higher in HK-2 EVs compared to the other two miRNAs as we found in urine samples. Although the origin of urinary exosomes is complex, it is possible that both proximal tubule and mesangial cells contribute to the increased expression of the three miRNAs in urinary exosomes.

There are several limitations of this study. First, relatively small numbers of patients were included; a study with a larger cohort is required. Second, renal biopsy was not performed, and serum exosomal miRNAs levels were not measured. Although another study reported that blood miRNA expression is reduced in patients with mild renal impairment, we could not determine whether the decrease in EV miRNA expression was caused by blood EV miRNAs or by decreased renal miRNA levels, as previously reported. Further studies are needed to explore the correlation between renal function and miR-192 levels in renal biopsies, blood, and urine at different stages of DN.

The expression of urinary EV miR-192, miR-194, and miR-215 was increased in T2DM patients with microalbuminuria. The combination of urinary EV miR-192 and TGF-*β*1 expression may provide new insight into the pathology of early-stage DN. The underlying mechanism relating urinary EV miRNAs and DN requires further exploration.

## Figures and Tables

**Table 1:** Demographic characteristics of subjects.

	Normo. (*N* = 30)	Micro. (*N* = 30)	Macro. (*N* = 20)
Age (years)	57.90 ± 1.50	59.00 ± 1.84	55.00 ± 1.81
Sex, male/female	27/3	27/3	17/3
Duration of diabetes (years)	7.08 ± 1.15	8.74 ± 1.26	8.33 ± 1.65
BMI	24.76 ± 0.61	24.26 ± 0.48	25.47 ± 0.61
SBP (mmHg)	136 ± 4.17	139.93 ± 3.22	149.8 ± 5.03^a^
DBP (mmHg)	82.23 ± 1.94	85.67 ± 2.16	90.5 ± 3.29^a^
HbA1C, %	9.43 ± 0.42	9.50 ± 0.34	9.47 ± 0.38
Scr (*μ*mol/L)	74.47 ± 2.72	78.87 ± 3.35	91.1 ± 8.11
Cystatin (mg/L)	0.98 ± 0.04	1.22 ± 0.08^a^	1.44 ± 0.12^a^
TC (mmol/L)	4.87 ± 0.16	4.75 ± 0.17	4.53 ± 0.21
TGs (mmol/L)	1.31 ± 0.09	1.67 ± 0.20	2.82 ± 0.40^ab^
HDL-C (mmol/L)	1.00 ± 0.03	0.97 ± 0.24	0.99 ± 0.26
LDL-C (mmol/L)	3.11 ± 0.16	3.08 ± 0.17	2.75 ± 0.17
eGFR (mL/min/1.73 m^2^)	94.78 ± 2.86	87.45 ± 3.57	86.82 ± 5.96
UACR (mg/mmol)	0.98 ± 0.10	17.07 ± 3.44^a^	210.02 ± 49.49^ab^
UAER (mg/24 h)	9.44 ± 0.74	145.47 ± 14.42^a^	1742.8 ± 371.14^ab^

Data are expressed as means ± SE. Normo.: normoalbuminuria; micro.: microalbuminuria; macro.: macroalbuminuria; BMI: body mass index; SBP: systolic blood pressure; DBP: diastolic blood pressure; HbA1C: glycosylated hemoglobin; Scr: serum creatinine; TC: cholesterol; TGs: triglycerides; HDL-C: high-density lipoprotein-cholesterol; LDL-C: low-density lipoprotein-cholesterol; eGFR: estimated glomerular filtration rate; UACR: urinary albumin/creatinine ratio; UAER: urinary albumin excretion rate. *P* values were calculated by Student's *t*-test. ^a^ *P* < 0.05 versus normo. group; ^b^ *P* < 0.05 versus micro. group.

**Table 2:** Levels of miRNAs in urinary EVs between groups.

miRNAs	Control	Normo.	Micro.	Macro.
miR-192	0.50 ± 0.41	1.06 ± 0.44	7.00 ± 1.71^ab^	2.93 ± 1.94^c^
miR-194	0.0021 ± 0.0012	0.0304 ± 0.0.0151	0.2074 ± 0.1191^ab^	0.0487 ± 0.0301
miR-215	0.0025 ± 0.0016	0.0308 ± 0.0232	0.1084 ± 0.0407^ab^	0.0151 ± 0.0084^c^

Data are expressed as means ± SE. Normo.: normoalbuminuria; micro.: microalbuminuria; macro.: macroalbuminuria. ^a^ *P* < 0.05 versus control group; ^b^ *P* < 0.05 versus normo. group; ^c^ *P* < 0.05 versus micro. group. *P* values were calculated by Mann-Whitney *U* test.

**Table 3:** Correlations between urinary EV miRNAs levels and renal function parameters in T2DM patients with normoalbuminuria (*n* = 30) and microalbuminuria (*n* = 30).

	miR-192		miR-194		miR-215	
	*r*	*P*	*r*	*P*	*r*	*P*
Scr	−0.018	0.874	−0.062	0.636	−0.062	0.584
Cystatin	−0.23	0.836	−0.02	0.88	−0.053	0.642
eGFR	0.104	0.360	0.131	0.322	0.139	0.220
